# A method for simultaneously determining both the inhalable fraction and vapor concentration to assess worker exposure to tri-*n*-butyl phosphate

**DOI:** 10.1093/joccuh/uiaf063

**Published:** 2025-10-30

**Authors:** Akito Takeuchi, Ai Yamada, Tomiko Tashiro, Maika Inoue, Yuriko Miyama, Kenta Ishii, Shinobu Yamamoto, Yoko Endo, Ginji Endo

**Affiliations:** Kinki Osaka Safety and Health Service Center, Japan Industrial Safety and Health Association, 2-3-8 Tosabori, Nishi-Ku, Osaka 550-0001, Japan; Occupational Health Research and Development Center, Japan Industrial Safety and Health Association, 5-35-2 Shiba, Minato-Ku, Tokyo 108-0014, Japan; Kinki Osaka Safety and Health Service Center, Japan Industrial Safety and Health Association, 2-3-8 Tosabori, Nishi-Ku, Osaka 550-0001, Japan; Department of Pathophysiological Laboratory Sciences, Nagoya University Graduate School of Medicine, 1-1-20 Daiko-minami, Higashi-Ku, Nagoya 461-8673, Japan; Kinki Osaka Safety and Health Service Center, Japan Industrial Safety and Health Association, 2-3-8 Tosabori, Nishi-Ku, Osaka 550-0001, Japan; Kinki Osaka Safety and Health Service Center, Japan Industrial Safety and Health Association, 2-3-8 Tosabori, Nishi-Ku, Osaka 550-0001, Japan; Kinki Osaka Safety and Health Service Center, Japan Industrial Safety and Health Association, 2-3-8 Tosabori, Nishi-Ku, Osaka 550-0001, Japan; Kanto Regional Safety and Health Service Center, Japan Industrial Safety and Health Association, 1st fl, Sigma Bldg, 3-7-12 Shibaura, Minato-ku, Tokyo 108-0023, Japan; Laboratory of Environmental Toxicology and Carcinogenesis, School of Pharmacy, Nihon University, 7-7-1 Narashinodai, Funabashi, Chiba 274-8555, Japan; Research Center for Safety Science, Nagoya University, Furo-cho, Chikusa-ku, Nagoya 464-8601, Japan; Kinki Osaka Safety and Health Service Center, Japan Industrial Safety and Health Association, 2-3-8 Tosabori, Nishi-Ku, Osaka 550-0001, Japan; Kinki Osaka Safety and Health Service Center, Japan Industrial Safety and Health Association, 2-3-8 Tosabori, Nishi-Ku, Osaka 550-0001, Japan

**Keywords:** air sampling method, gas chromatography, personal exposure monitoring, tri-*n*-butyl phosphate, workplace air

## Abstract

**Objectives**: This study aimed to develop a personal exposure measurement method that concurrently determines both the inhalable fraction and vapor concentration of tri-*n*-butyl phosphate (TBP).

**Methods**: A personal sampler, the IFV Pro, equipped with a glass-fiber filter (GFF) and a Chromosorb 106 adsorption tube was used. Postsampling, TBP was extracted or desorbed separately from the GFF and Chromosorb 106 using dichloromethane containing tri-*n*-amyl phosphate as an internal standard. The solutions obtained were analyzed via gas chromatography with a flame ionization detector. The evaluation parameters for validating the method included extraction efficiency, desorption efficiency, retention efficiency, storage stability, method quantitation limit, and reproducibility.

**Results**: The extraction efficiency of TBP from the GFF ranged from 97% to 100%, whereas the desorption efficiency from Chromosorb 106 was between 98% and 102%. The retention efficiencies for TBP were 0% (not quantitative, <0.68 μg/sample) to 88% on the GFF, and 7% to 95% on Chromosorb 106, culminating in a total retention efficiency of 95%-98%. Relative SDs, indicative of reproducibility, ranged from 0.8% to 6.9%. Both TBP on the GFF and in the Chromosorb 106 tube maintained stability under refrigeration at 4°C for at least 7 days. The method quantitation limit was established at 6.00 μg/sample.

**Conclusions**: A method was established to measure both the inhalable fraction and vapor concentration of TBP across an air concentration range of 0.05 to 10 mg/m^3^. This method is potentially valuable for assessing TBP exposure levels in workers.

## Teaser

Key points:

- **What is already known on this topic:** Tri-*n*-butyl phosphate (TBP) is used in various industrial applications. Owing to its physical properties, TBP can exist both as an aerosol and a vapor in workplace environments. Despite this, there has been no adequate method to simultaneously quantify both states to evaluate worker exposure to TBP.
- **What this study adds:** This research introduces a method using an IFV Pro Sampler equipped with a glass-fiber filter and a Chromosorb 106 adsorption tube. The method enables the measurement of personal exposure concentrations of TBP ranging from 0.05 to 10 mg/m^3^.
- **How this study might affect research, practice, or policy:** The developed method provides a means to more accurately estimate TBP exposure levels among workers, potentially influencing occupational safety standards and health policies.

## 1. Introduction

Tri-*n*-butyl phosphate (TBP, CAS RN: 126-73-8) is a colorless, odorless liquid widely used in various industries.^1^^,^^2^ It serves as a solvent for the extraction and purification of rare earth elements such as platinum and uranium, an antifoaming agent in the textile and paper industries, a plasticizer or solvent in the production of plastics, vinyl resins, and natural rubbers, and as a component in aircraft hydraulic fluids.^1-3^

In Japan, employers engaged in the manufacture or handling of TBP must conduct a risk assessment under the Industrial Safety and Health Act (Article 57-3).^4^ Although the Japan Society for Occupational Health has not set an occupational exposure limit (OEL) for TBP, the American Conference of Governmental Industrial Hygienists (ACGIH)^1^ has proposed an OEL of 5 mg/m^3^ (threshold limit value–time-weighted average, TLV-TWA) as “inhalable fraction and vapor (IFV).” Moreover, the Deutsche Forschungsgemeinschaft (DFG)^5^ has proposed an OEL of 11 mg/m^3^ (Maximale Arbeitsplatzkonzentrationen, MAK), noting that “this substance can occur simultaneously as vapor and aerosol.” Owing to the limited human data on TBP exposure, these OELs are derived from animal studies and are intended to prevent bladder epithelium irritation—which can cause necrosis, hyperplasia, and subsequent neoplasia—as well as irritation of the skin, eyes, and upper respiratory tract.^1^ At these OELs, the estimated saturated vapor concentration of TBP can substantially contribute to exposure, and TBP particulates captured on sampling media may be lost to evaporation.^1^ Consequently, risk assessments for TBP exposure require determination of TBP concentrations in both the particle and vapor phases.

Current literature indicates a few methods for measuring personal exposure to TBP.^2^^,^^6^^,^^7^ However, none can simultaneously assess concentrations in both the particle and vapor phases within the concentration ranges pertinent to the TLV-TWA and MAK values used for risk assessments. Thus, our study aimed to develop a method capable of measuring personal exposure to TBP, specifically employing the TLV-TWA value, given its lower threshold compared with the MAK value.

## 2. Materials and methods

### 2.1. Materials

TBP and tri-*n*-amyl phosphate (TAP, CAS RN 2528-38-3) were sourced from Tokyo Chemical Industry (TBP, Product No. P0266, >99.0% purity; TAP, Product No. P0265, >98.0% purity). Dichloromethane (DCM, guaranteed reagent grade, Product No. 10158-00) was obtained from Kanto Chemical Co, Inc. According to the method described by Solbu et al,^7^ DCM containing 200 μg/mL TAP as an internal standard was used for extraction and desorption. TBP standard solutions were prepared in DCM and stored at 4°C.

### 2.2. Analytical procedure and instrumental conditions

Postsampling, the glass-fiber filter (GFF, 25 mm in diameter, Product No. AP2002500, Merck Millipore Ltd) and each section of the Chromosorb 106 sorbent tube (75 mg/35 mg, Product No. 226-49-106, SKC Inc) were placed into separate glass test tubes, with the front glass wool plug of Chromosorb 106 combined with its corresponding front sorbent section. Each tube received 3 mL of extraction/desorption solvent (DCM/TAP mixture) followed by sonication for 30 min. The extracted or desorbed solutions were then transferred to autosampler vials for gas chromatographic analysis.

The samples were analyzed using a gas chromatograph (6890 N) equipped with a flame ionization detector (GC/FID, Agilent Technologies). The GC/FID system used an HP-5 column (30 m × 0.25 mm ID, 0.25 μm film thickness, Agilent Technologies). The operational settings were as follows: inlet and detector temperatures at 280°C; injection volume of 1 μL; pulsed split injection with a split ratio of 20:1, pulse pressure of 25 psi, and pulse time of 1 minute. The carrier gas (helium) flow rate was set at 1.0 mL/min. The column temperature protocol began at 100°C, held for 1 minute, then ramped to 280°C at a rate of 10°C/min.

### 2.3. Method validation procedure

The sampling process involved the IFV Pro Sampler (Product No. 225-49, SKC Inc), which incorporated a GFF and a downstream Chromosorb 106 sorbent tube. An AirChek CONNECT sampling pump (Product No. 220-4000, SKC Inc) maintained the flow at 1 L/min, as recommended by the manufacturer,^8^ with a maximum sampling duration of 2 hours, consistent with the methods used in Solbu et al’s study.^7^ Method development and validation adhered to guidelines from the National Institute for Occupational Safety and Health (NIOSH)^9^ and the US Department of Labor, Occupational Safety and Health Administration (OSHA).^10^ The procedure for extraction/desorption efficiency testing was as follows: the front glass wool plug of the Chromosorb 106 tube and the GFF were each spiked with 5 μL of a TBP standard solution at 4 different concentrations (1.2, 12, 120, and 240 mg/mL). The spiked GFF was allowed to dry completely at room temperature for 10 minutes, while the spiked Chromosorb 106 tube was exposed to room air for the same duration. For retention efficiency and storage stability tests, the GFF was spiked using the same method. The spiked GFF and an unspiked Chromosorb 106 tube were assembled in the IFV Pro Sampler, through which room air (temperature 20.9-24.1°C; relative humidity 27%-59%) was drawn for 2 hours. To assess storage stability, the GFF was sealed within its filter cassette, the Chromosorb 106 tube was capped, and both were refrigerated at 4°C for 7 days. The spiking levels of TBP used for these tests varied: 4 levels (6.00, 60.0, 600, and 1200 μg) for retention efficiency, and 3 levels (6.00, 60.0, and 1200 μg) for storage stability tests. The spiked amounts used for the retention efficiency test (6.00, 60.0, 600, and 1200 μg) correspond to air concentrations of approximately 0.05, 0.5, 5, and 10 mg/m^3^, respectively, representing approximately 1/100, 1/10, 1, and 2 times the TLV-TWA value. To obtain a calibration curve, TBP standard solutions were added to the extraction/desorption solvents to achieve 8 concentrations—1, 2, 10, 20, 100, 200, 300, and 400 μg/mL—and then analyzed. Calibration curves were constructed by plotting the peak area ratio of TBP to TAP versus their respective concentrations.

## 3. Results

**Figure 1:**
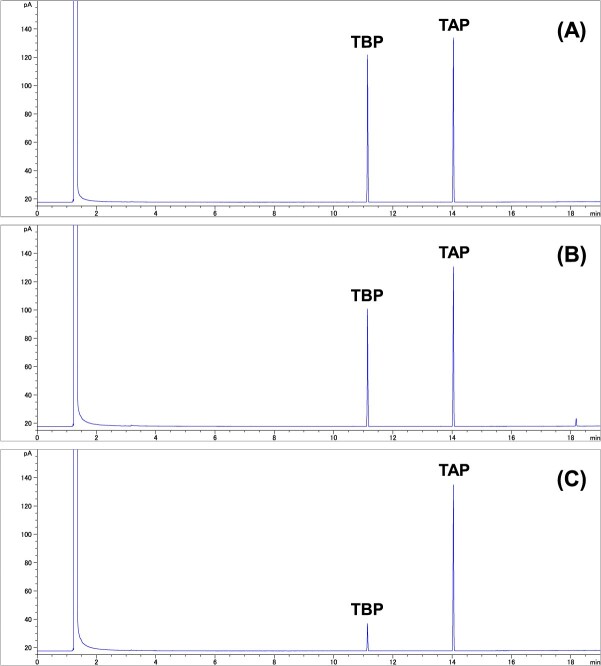
Chromatograms of (A) a standard solution of tri-*n*-butyl phosphate (TBP, 200 μg/mL), (B) glass-fiber filter (TBP, 159 μg/mL), and (C) front sorbent section of Chromosorb 106 (TBP, 36 μg/mL) in the retention efficiency test. The test used an IFV Pro Sampler fitted with a glass-fiber filter spiked with 600 μg TBP and an unspiked Chromosorb 106 tube; 120 L of room air was drawn. Tri-*n*-amyl phosphate (TAP) served as the internal standard in the analysis.

The method validation results are summarized in Table 1. The extraction efficiency of TBP from the GFF ranged from 97% to 100%, and the desorption efficiency from Chromosorb 106 ranged from 98% to 102%, demonstrating high reproducibility. Retention efficiencies were determined by comparing the amount of TBP spiked onto each sampler with the amount recovered after drawing room air through it, indicating the ability of each sampler to retain TBP. Retention efficiencies were 0% (not quantitative, <0.68 μg/sample) to 88% for GFF, and 7% to 95% for Chromosorb 106, with a combined total retention efficiency of 95%-98%. The relative SDs for the retention efficiency ranged from 0.8% to 6.9%. No TBP was detected on the back sorbent section of any spiked Chromosorb 106 tube during the retention efficiency test, indicating no occurrence of breakthrough. Chromatograms of a 200 μg/mL TBP standard solution, the solution extracted from the GFF spiked with 600 μg TBP after drawing 120 L of room air, and the desorbed solution from the unspiked Chromosorb 106 tube after the same air volume sampling are shown in Figure 1a-c, respectively, with corresponding concentrations of 159 μg/mL and 36 μg/mL for Figure 1b and c. No TBP was detected on either the GFF or Chromosorb 106 when the IFV Pro Sampler drew only room air without spiked TBP. Storage stability tests evaluated TBP stability on the GFF and within the Chromosorb 106 tube by comparing the total TBP recovered after storage to that measured immediately postsampling. Over a storage period of 3-7 days, TBP recovery rates at spiked levels of 6.00 μg, 60.0 μg, and 1200 μg varied between 96% and 108%, 95% and 99%, and 92% and 100%, respectively. The calibration curve was linear over a range of 1-400 μg/mL, with a coefficient of determination (*R*^2^) of 0.999. The instrumental quantification limit, calculated as 10 times the SD (*n* = 5) of the peak area ratio (TBP/TAP) of the lowest standard solution (1 μg/mL) divided by the slope of the calibration curve, was 0.68 μg/sample. The method quantification limit, defined as the minimum spiked amount of TBP yielding greater than 90% retention efficiency, was established at 6.00 μg/sample.

**Table 1:** Extraction, desorption, and retention efficiency tests (each *n* = 5).

**Spiked amount, μg**	**EE,** ^**a**^ **%**		**DE,** ^**b**^ **%**		**RE,** ^**c**^ **%**					
**GFF**			**Chromosorb 106**		**GFF**		**Chromosorb 106**		**Total**	
	**Mean ± SD**	**RSD**	**Mean ± SD**	**RSD**	**Mean ± SD**	**RSD**	**Mean ± SD**	**RSD**	**Mean ± SD**	**RSD**
**6.00**	99 ± 2.7	2.7	98 ± 1.7	1.7	NQ	—	95 ± 6.6	6.9	95 ± 6.6	6.9
**60.0**	99 ± 1.1	1.1	100 ± 3.1	3.1	5 ± 0.6	13.0	91 ± 2.1	2.3	96 ± 2.3	2.4
**600**	100 ± 1.9	1.9	102 ± 1.7	1.6	81 ± 0.5	0.6	17 ± 1.1	6.3	98 ± 0.8	0.8
**1200**	97 ± 3.0	3.1	98 ± 2.6	2.6	88 ± 1.7	1.9	7 ± 0.9	12.8	95 ± 2.3	2.4

Abbreviations: DE, desorption efficiency; EE, extraction efficiency; GFF, glass-fiber filter; NQ, not quantitative (<0.68 μg/sample); RE, retention efficiency; RSD, relative standard deviation.

a The sample for the extraction efficiency test was prepared by spiking the GFF with TBP and left at room temperature for 10 minutes to dry completely.

b The sample for desorption efficiency test was prepared by spiking Chromosorb 106 with TBP and passing it through room air at a sampling flow rate of 1 L/min for 10 minutes.

c The sample for the retention efficiency test was prepared by setting the TBP spiked GFF and the Chromosorb 106 tube without TBP spiked in the IFV Pro Sampler, and then room air was pulled through the sampler at a sampling flow rate of 1 L/min for 2 hours. The spiked amounts correspond to air concentrations of approximately 0.05–10 mg/m^3^.

## 4. Discussion

Among the evaluated methods, the NIOSH method,^6^ which uses a mixed cellulose ester membrane filter, is unable to measure TBP concentrations in the vapor phase. In contrast, the approaches by Rosenberger and Bader^2^ and Solbu et al,^7^ which employ a combination of a GFF and an adsorbent tube, can assess TBP concentrations in both the particle and vapor phases. Given the requirements for comprehensive risk assessment of worker exposure to TBP, the methods by Rosenberger and Bader and Solbu et al are more suitable. However, these methods were tested at concentration ranges significantly lower than the TLV-TWA. Consequently, we refined Solbu et al’s methodology to extend its applicability to the TLV-TWA range. Our adaptation used the GFF and Chromosorb 106 tube as sampling materials within the IFV Pro Sampler setup. The IFV Pro Sampler is designed based on the IOM inhalable sampling head (Institute of Occupational Medicine, Edinburgh, UK); this sampler operates at a lower flow rate to accommodate the addition of a sorbent tube. This configuration allows the GFF to capture the inhalable fraction of TBP while the Chromosorb 106 adsorbs the vapor phase. Furthermore, the results of the retention efficiency test suggested that some TBP initially collected on the GFF vaporizes during the sampling and is subsequently captured by the Chromosorb 106. The validation results indicated that the sampling performance of the proposed method has robust reproducibility, and it also confirmed that TBP on both the GFF and in the Chromosorb 106 tube can be effectively refrigerated at 4°C for at least 7 days, supporting the feasibility of this sampling method for realistic field conditions.

Whereas Rosenberger and Bader^2^ and Solbu et al^7^ employed a mass spectrometer (MS) as the detector, Rosenberger and Bader observed nonlinear calibration curves within their investigated concentration range. This finding was consistent with our preliminary experiments at air concentrations ranging from 1/100 to 2 times the TLV-TWA value proposed by the ACGIH. Consequently, we opted for an FID, which demonstrated promising results: the air concentration range of TBP measurable by our method spans 0.05-10 mg/m^3^ in a 2-hour sampling period with a sampling air volume of 120 L, aligning with 1/100 to 2 times the TLV-TWA value.

This study has some limitations. Primarily, the experiment used samplers spiked with TBP standard solutions, as continuous generation of standard aerosols and vapors at known concentrations was not feasible. This issue is common in studies developing air sampling methods for chemical substances and represents a significant methodological challenge. Additionally, the method was validated under typical laboratory conditions. Although temperature and humidity may differ between typical laboratory conditions and actual workplaces, their effects on the evaluation parameters have not been investigated. Moreover, the use of an FID, despite facilitating a linear calibration curve, lacks the selectivity of MS. This could lead to potential interference from substances sharing the same retention time as TBP, although adjustments in chromatographic conditions might mitigate such interference.

## 5. Conclusions

We successfully developed a method capable of simultaneously determining the IFV concentration of TBP in air. This method enables the measurement of personal exposure concentrations of TBP ranging from 0.05 to 10 mg/m^3^, covering 0.01-2 times the TLV-TWA value proposed by the ACGIH. It is therefore a valuable tool for assessing the exposure levels of workers to TBP.

## Data Availability

Data underlying this article will be made available upon reasonable request to the corresponding author.
